# The Role of Ciliate Protozoa in the Rumen

**DOI:** 10.3389/fmicb.2015.01313

**Published:** 2015-11-26

**Authors:** Charles J. Newbold, Gabriel de la Fuente, Alejandro Belanche, Eva Ramos-Morales, Neil R. McEwan

**Affiliations:** ^1^Institute of Biological Environmental and Rural Sciences, Aberystwyth University, Aberystwyth, UK; ^2^Departament de Producció Animal, Escola Tècnica Superior d’Enginyeria Agrària, Universitat de Lleida, Lleida, Spain

**Keywords:** rumen, protozoa, defaunation, methane, microbial diversity

## Abstract

First described in 1843, Rumen protozoa with their striking appearance were assumed to be important for the welfare of their host. However, despite contributing up to 50% of the bio-mass in the rumen, the role of protozoa in rumen microbial ecosystem remains unclear. Phylogenetic analysis of 18S rDNA libraries generated from the rumen of cattle, sheep, and goats has revealed an unexpected diversity of ciliated protozoa although variation in gene copy number between species makes it difficult to obtain absolute quantification. Despite repeated attempts it has proven impossible to maintain rumen protozoa in axenic culture. Thus it has been difficult to establish conclusively a role of ciliate protozoa in rumen fiber degradation. The development of techniques to clone and express ciliate genes in λ phage, together with bioinformatic indices to confirm the ciliate origin of the genes has allowed the isolation and characterization of fibrolytic genes from rumen protozoa. Elimination of the ciliate protozoa increases microbial protein supply by up to 30% and reduces methane production by up to 11%. Our recent findings suggest that holotrich protozoa play a disproportionate role in supporting methanogenesis whilst the small *Entodinium* are responsible for much of the bacterial protein turnover. As yet no method to control protozoa in the rumen that is safe and practically applicable has been developed, however a range of plant extract capable of controlling if not completely eliminating rumen protozoa have been described.

## Introduction

First described in 1843, Rumen protozoa with their striking appearance were assumed to be important for the welfare of their host. However, despite the fact that protozoa can contribute up to 50% of the bio-mass in the rumen, the role of protozoa in rumen microbial ecosystem remains unclear (Williams and Coleman, 1992). Here we evaluate recent information on the role of ciliate protozoa in the rumen microbial ecosystem.

## Diversity and Phylogeny of Rumen Ciliate Protozoa

Since they were first discovered by Gruby and Delafond (1843), studies on rumen protozoa have relied on morphologic identification by optical microscopy. There are currently no culture collections of rumen ciliates, so researchers have to use photomicrographs for identification (Ogimoto and Imai, 1981; Williams and Coleman, 1992) or line drawings (Dogiel, 1927; Kofoid and MacLennan, 1930, 1932, 1933; Latteur, 1968, 1969, 1970; Dehority, 1993). It is widely accepted that microscopic identification and counting represents the gold standard for analyzing ciliate community structure in rumen studies (Williams and Coleman, 1992). However, the polymorphic nature of these microbes (Dehority, 1994, 2006) requires a high level of experience by the researcher to identify rumen ciliates at a genera or species level (Dehority, 2008). Thus, alternative ways to identify protozoa are needed since microscopic techniques are laborious and highly demanding.

Early molecular studies on rumen ciliate protozoa focussed on sequencing of 18S rRNA genes to clarify the internal phylogeny of the class Litostomatea (Embley et al., 1995; Wright et al., 1997). Embley et al. (1995) were the first to describe the monophyletic position of the anaerobic rumen ciliates *Dasytricha ruminantium* and *Polyplastron multivesiculatum* based on 18S rRNA genes and Wright et al. (1997), Wright and Lynn (1997a,b) provided molecular data for *Diplodinium dentatum*, *Entodinium caudatum*, *Epidinium caudatum*, *Eudiplodinium maggii*, *Isotricha intestinalis*, *Ophryoscolex purkynjei*, and *Polyplastron multivesiculatum*.

Numerous qualitative and quantitative studies focused on the diversity of ciliates have been performed since then; these studies have used a variety of techniques, including restriction fragment length polymorphism (RFLP; Regensbogenova et al., 2004a, Tymensen et al., 2012b), denaturing gradient gel electrophoresis (DGGE; Regensbogenova et al., 2004b, McEwan et al., 2005; de la Fuente et al., 2009; Belanche et al., 2010a, Kittelmann and Janssen, 2011), real-time PCR (qPCR; Sylvester et al., 2004; Skillman et al., 2006; Belanche et al., 2010a, Kittelmann and Janssen, 2011), fluorescence in situ hybridization (FISH; Xia et al., 2014) and next generation sequencing (Kittelmann et al., 2013, 2015; Ishaq and Wright, 2014; Moon-van der Staay et al., 2014).

Microscopy holds several advantages over PCR-based molecular methods for studying ciliate protozoa. First, while the vast majority of intestinal ciliates have been characterized morphologically, there is a lack of 18S rRNA gene reference sequences for many of the observed genera and species. Second, as discussed below, copy number variation of ribosomal RNA genes across the different genera or under different growth conditions may skew the observed proportions of these genera in a sample (Medinger et al., 2010). Studies using 18S rRNA gene surveys reveal an apparent higher diversity of ciliates than estimate by conventional morphological methods (Moon-van der Staay et al., 2014).

Like other ciliates, rumen protozoa contain two kinds of nuclei: a micronucleus and a macronucleus. The micronucleus possesses clearly visible chromosomes, is diploid, and synthesizes only a trace of RNA. The macronucleus contains no discernible chromosomes, has many times the diploid amount of DNA, divides amitotically, and provides virtually all of the RNA needed to run the vegetative life of the cell (Prescott and Murti, 1974). The extremely high copy number of rDNA in the macronuclear genome of ciliates, as previously found (Gong et al., 2013), is understandable considering the life history, large cell size and rapid growth of these organisms (Cavalier-Smith, 2005; Zhu et al., 2005; Godhe et al., 2008). In rumen ciliates, while the small protozoa such as *Entodinium*, tended to be under-represented, larger protozoa such as *Epidinium* or *Polyplastron* tended to be over-represented by a pyrosequencing approach compared to microscopic enumeration (Kittelmann et al., 2015). Similarly, *Epidinium caudatum*, which is approximately five times larger by volume than *Entodinium caudatum* (Dehority, 1993) also has approximately five times more 18S rRNA gene copies encoded in its genome (Sylvester et al., 2009). These results agree with the finding that PCR-based methods return lower estimates of abundance of small *Entodinium* spp. (Tymensen et al., 2012a) and overestimates of the abundance of, e.g., *Polyplastron* spp. (Ishaq and Wright, 2014). Knowing the copy numbers and the variations of rDNA sequences within individual eukaryotes is important both for interpreting rDNA-based diversity surveys and when 18s rDNA is used to quantify protozoal biomass. (Crosby and Criddle, 2003; Thornhill et al., 2007; Herrera et al., 2009; Amaral-Zettler et al., 2011). To date no studies addressing the number of copies per cell and variations of rDNA in rumen protozoa have been published. This is especially true as rDNA-based barcoding and microbial diversity studies using high-throughput sequencing are becoming more popular (Amaral-Zettler et al., 2009) and molecular tools based on marker gene surveys are now widely used to study the diversity of other microbes (Schlötterer, 2004; Case et al., 2007; Langille et al., 2013). Studies in non-rumen ciliates have shown that the rDNA copy number variation between and within ciliate species highlighting the difficulty of using the rDNA sequence number-based approach to infer the relative abundance of microbial eukaryotic cells in environmental samples (Wintzingerode et al., 1997; Medinger et al., 2010). Thus, latest methods based on 18S rRNA genes may be unreliable when estimating α-diversity or relative abundances of different genera and species in a given sample, although they can, reliably determine trends in relative abundances of genera and species between different samples (β-diversity). More research comparing molecular and traditional methods is needed (de la Fuente et al., 2009; Tymensen et al., 2012b; Kittelmann et al., 2015).

## Functional Genes in Rumen Ciliate Protozoa

Although there are numerous copies of rDNA in ciliate macronucleus, it is likely that only a small portion of these genes are transcriptionally active in ciliates under any given growth condition, as previously shown for other eukaryotes (Reeder, 1999). Studies in *Tetrahymena thermophila*, *Paramecium tetraurelia*, and *Oxytricha trifallax* have pinpointed the important role of non-coding RNAs in genome rearrangement events (Feng and Guang, 2013). In spirotrichous ciliates, such as *Euplotes*, *Stylonychia*, and *Oxytricha*, more than 95% of the micronuclear DNA is eliminated to form the macronucleus during sexual reproduction (Swart et al., 2013) possible as a mechanism to allow the cell to adapt during times of stress. Moreover, in the macronucleus, the remaining genome is severely fragmented and these fragments are sorted and reordered under the guidance of transcripts from the parental macronucleus to produce protein-coding genes (Nowacki et al., 2011). None of these processes have been studied so far in rumen protozoa, due to the difficulties in getting full length genomes of rumen ciliates.

Despite repeated attempts it has proven impossible to maintain rumen protozoa in axenic culture. Thus it has been difficult to establish conclusively a role of ciliate protozoa in the rumen and specifically fiber degradation. Early studies sought to isolate and characterize cellulose, hemicellulase and xylanase enzymes from washed protozoal preparations (Howard et al., 1960; Bailey et al., 1962; Clayet et al., 1992), however the presence of both extra and intra cellular symbiotic bacteria in protozoal preparations made it difficult to be sure that the isolated activity was truly of protozoal origin (Delfosse-Debusscher et al., 1979; Thines-Sempoux et al., 1980). We developed techniques to clone and express ciliate genes in λ phage (Eschenlauer et al., 1998), using FISH to confirm the protozoal identity of the expressed genes (Newbold et al., 2005) and developing bioinformatic indices to confirm the ciliate origin of the genes (McEwan et al., 2000). Using these techniques we and others have been able to isolate and characterize genes from a range of rumen protozoa (McEwan et al., 1999; Newbold et al., 2005; Belzecki et al., 2007; Boxma et al., 2007). This includes a wide range of fibrolytic enzymes a number of which have been found to contain multiple domains with binding domains and putative chimeric constructs being observed suggesting a highly evolved fibrolytic capacity in the rumen ciliates (Devillard et al., 1999, 2003; Takenaka et al., 1999, 2004; Wereszka et al., 2004; Bera-Maillet et al., 2005). This observation has been confirmed by recent metagenomic screening of protozoal glucosidases and eukaryotic metatranscriptomes that have confirmed that a diverse range of diverse glycoside hydrolases are present in the rumen protozoa (Findley et al., 2011; Qi et al., 2011).

Based on large-scale construction and analysis of phylogenies of over 4000 Expressed Sequence Tag libraries from the rumen ciliates *Entodinium caudatum*, *Eudiplodinium maggii*, *Metadinium medium*, *Diploplastron affine*, *Polyplastron multivesiculatum*, *Epidinium ecaudatum*, *Isotricha prostoma*, *Isotricha intestinalis*, and *Dasytricha ruminantium*. Ricard et al. (2006) concluded there was extensive evidence of horizontal gene transfer (HGT; 148 out of 3563 non-redundant genes) from bacteria and archaea in rumen ciliate genomes. Among the HGT candidates, they reported an over-representation (>75%) of genes involved in metabolism, specifically in the catabolism of complex carbohydrates (Ricard et al., 2006), suggesting that HGT may have been important in allowing rumen ciliates to adapt to new niches within the rumen and that fibrolytic genes were acquired by protozoan from bacterial sources (Findley et al., 2011).

## Role of Protozoa in the Rumen

Despite the fact that that protozoa make up a large portion of the rumen biomass, their role in ruminal fermentation and their contribution to the metabolism and nutrition of the host is still an area of substantial controversy (Williams and Coleman, 1992). In the last section of this paper we will review different strategies to manipulate the rumen protozoal density; however most of these dietary interventions also lead to modifications in rumen function making it difficult to assess the effect of the rumen protozoa *per se*. Rumen protozoa are not essential to the animal to survive and defaunation (the removal of protozoa from the rumen using a wide variety of chemicals and physical techniques) and protozoa-free animals have been used to study the role of ciliate protozoa in the rumen function without been affected by dietary interventions (Williams and Coleman, 1992). This paper does not aim to conduct a complete review on the effect of defaunation but compiles the most relevant publications since the excellent and complete review by Williams and Coleman (1992). Thus, a meta-analysis was conducted to study the main effects of defaunation based on 23 *in vivo* studies comprising 48 comparisons (Kreuzer et al., 1986; Vermorel and Jouany, 1989; Ushida et al., 1990; Frumholtz, 1991; Nagaraja et al., 1992; Hegarty et al., 1994, 2008; Faichney et al., 1999; Chandramoni et al., 2001; Machmuller et al., 2003; Eugène et al., 2004; Ozutsumi et al., 2005; Ohene-Adjei et al., 2007; Yáñez-Ruiz et al., 2007a,b; Bird et al., 2008; Morgavi et al., 2008, 2012; Belanche et al., 2011, 2012b, 2015; Mosoni et al., 2011; Zhou et al., 2011). Most of the studies were performed using sheep (87%), while the rest used cattle (13%). Isolation of new born animals from their mothers (40%), use of detergents and other chemicals (35%, using sodium lauryl sulfate, alkanes, synperonic NP9, calcium peroxide, copper sulfate etc.), and ruminal manipulation (25%, emptying and washing of the rumen), were used in order to achieve defaunation of the animals. Trials where the effect of additives, other than defaunation, were significant were discarded from the meta-analysis. Similarly, studies using monofaunated animals or selective defaunation were not included. The majority of the data (75%) were from trials in which animals were fed at maintenance and the remaining trials were from production trials. Animals were mainly fed mixed diets (90%) composed of forages supplemented with concentrate, while 10% of the diets were purely composed of forage. Rumen fermentation data and methane emissions were reported in most trials (69%), while information about digestion (31%), animal performances (12%) and rumen microbial populations (10%) was less abundant. Methane production was measured in chambers (75% in open chambers or respiratory calorimeters) or with the SF_6_ (sulfur hexafluoride) tracer method (25% of studies).

Multiple comparisons were included from an individual publication with multiple studies. For each comparison included in the analysis, the effect size was calculated as the natural logarithm of the response ratio (mean value in the defaunated treatment divided by mean value in control treatment) and variance of the ratio calculated based on the reported standard deviation or standard error of the mean for each comparison (Viechtbauer, 2010). All defaunation effects were weighted according to the number of observations (*n*) in each comparison. The meta-analysis was computed fitting a random-effect model with a DerSimonian-Laird estimator (Dersimonian and Laird, 1989) for assessing heterogeneity (τ^2^) in the Metafor package of R for each category separately as follow:

$$\theta_{i}=\mu +e_{i}$$

where θ*_i_* = true effect size in the ith study, μ = overall true effect size and *e_i_* = random deviation from the overall effect size [*u_i_* ∼ N (0, τ^2^)] (Viechtbauer, 2010). For evaluating the response ratio, values below one indicated a negative, while values above one indicated a positive effect of defaunation on that particular parameter.

**FIGURE 1:**
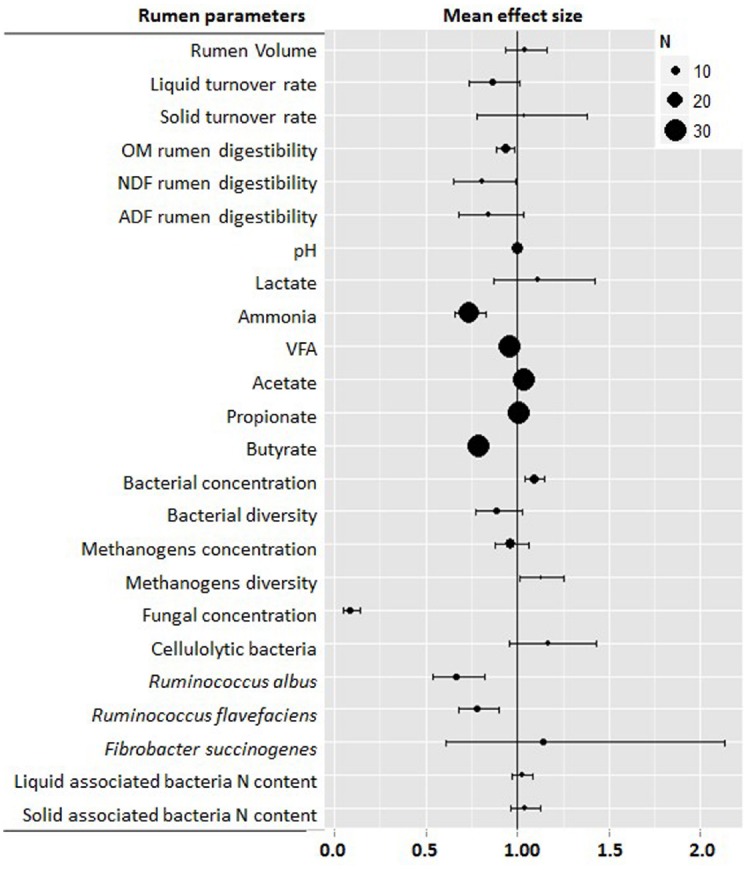
**Meta-analysis describing the effects of defaunation on rumen function.** For each parameter information is provided about the number of studies, observations (n), and range of values. Graph shows the mean effects and 95% confidential intervals. Values below one indicate a negative effect, while those above one indicate a positive effect of defaunation on that particular parameter.

**FIGURE 2:**
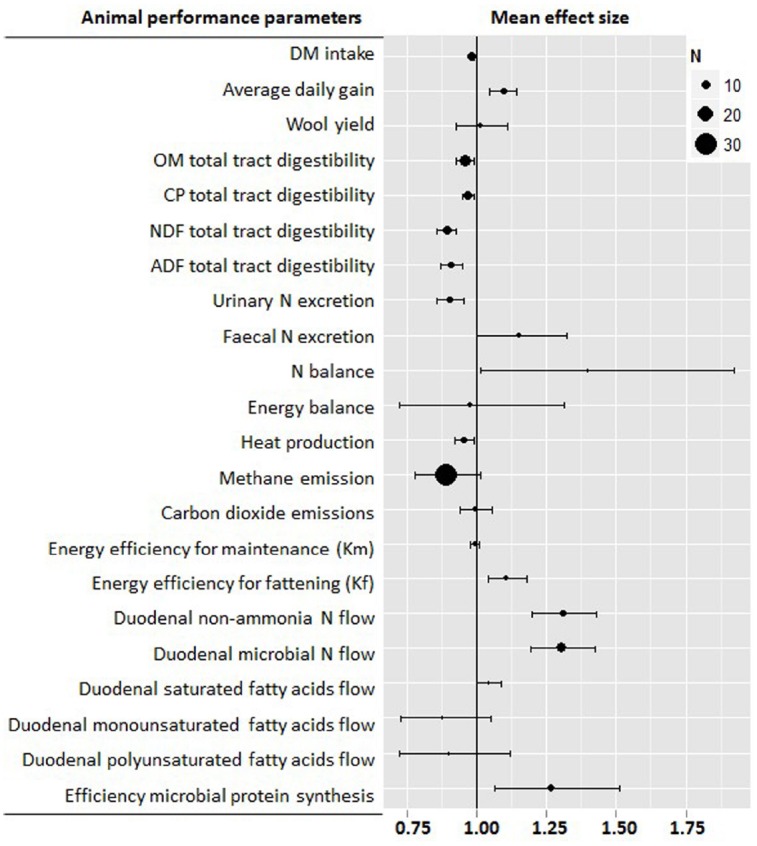
**Meta-analysis describing the effects of defaunation animal performance.** For each parameter information is provided about the number of studies, observations (n), and range of values. Graph shows the mean effects and 95% confidential intervals. Values below one indicate a negative effect, while those above one indicate a positive effect of defaunation on that particular parameter.

This meta-analysis confirmed many of the previously reported results (Williams and Coleman, 1992) and helped clarify number of areas in which results were still conflicting. Figure 1 shows the main effects of defaunation on the rumen function indicating that the physical and chemical characteristics of the rumen environment were changed by defaunation; although the nature of the observed change was not always consistent. Rumen volume and solid turnover rate were unaffected by defaunation, while the liquid turnover rate tended to decrease (–14%, *P* = 0.07). Elimination of protozoa from the rumen significantly decreased rumen OM digestibility (–7%, *P* = 0.008) and particularly NDF (–20%, *P* = 0.040) and ADF digestibility (–16%, *P* = 0.100), probably as a result of the loss of protozoal fibrolytic activity. This activity seems however to differ across the different protozoal groups: Large *Ophyroscolecidae* such as *Epidinium*, *Polyplastron* and *Eudiplodinium* have greater endoglucanase and xylanase activity (Williams and Coleman, 1992). On the other hand, *Entodinium* spp. have only weak activity (Williams and Coleman, 1992). Similarly, *Dasytricha* has glucosidase and cellobiosidase activity but negligible fibrolytic activity (Takenaka et al., 2004). The lower rumen digestibility in defaunated animals is partially compensated by a greater post ruminal digestion resulting in less pronounced differences in terms of total tract digestibility for OM (–4%, *P* = 0.089), CP (–3%, *P* = 0.034), NDF (–11%, *P* < 0.001), and ADF (–9%, *P* < 0.001; Figure 2). Another compensatory factor could be a shift toward more energetically efficient reactions in the rumen (less methane emissions) and less metabolic energy required by defaunated animals to eliminate the excess of urea as a result of the lower bacterial protein breakdown and ammonia levels in the rumen. Despite these compensatory mechanism, the decrease in feed digestibility is likely to be the main drawback of defaunation since it could limit the feed intake and efficiency of feed utilization at production levels of intake; this coupled to the lack of a commercially viable approach (see below) to defaunation, mean that defaunation is not recommended as a methane mitigation strategy under farm conditions (Hristov et al., 2013).

The total concentration and production of fermentation products also differs in faunated and defaunated animals. A decrease in rumen ammonia (–26%, *P* < 0.001) is probably the most consistent of the observed effects of protozoal elimination and seems to be due to decreased bacterial protein breakdown and feed protein degradability in the absence of rumen protozoa (Williams and Coleman, 1992). Moreover it has been demonstrated that although bacterial predation by rumen protozoa is dependent on the protozoal size, holotrich protozoa have a much lower predatory activity than entodiniomorphids (Belanche et al., 2012a) and ultimately a lower impact on rumen ammonia concentration (Belanche et al., 2015) and duodenal microbial protein flow (Ivan et al., 2000a; Ivan, 2009). However, this negative effect of entodiniomorphids on the duodenal protein flow may be overestimated due to the lack of a reliable marker to measure protozoal flow (Broderick and Merchen, 1992). In previous reviews higher lactate levels have been reported in defaunated animals (Williams and Coleman, 1992) because protozoa consume lactate more rapidly than bacteria (Newbold et al., 1986). Our meta-analysis showed a numerical increase in lactate concentration in defaunated animals (+11%) but this increase was non-significant possibly because the dataset used in this meta-analysis was mainly composed by high-forage diets which are less prone to promote these particular nutritional disorders.

Although rumen pH was unaffected, the lower VFA concentration observed in defaunated animals (–5%, *P* = 0.013) seems to highlight the role of protozoa in the synthesis of VFA and feed degradation. The ability of protozoa to engulf exogenous fatty acids (Karnati et al., 2009) may divert more carbon toward VFA production in preference to fatty acid synthesis and ultimately increase VFA production. More interestingly, our data suggested that defaunation substantially decreased butyrate (–22%, *P* < 0.001), slightly increased acetate (+3%, *P* < 0.001) and had no effect on propionate molar proportions. In a previous meta-analysis (Noziere et al., 2011) it has been reported that NDF digestibility positively correlates with acetate molar proportion (*r* = 0.95) but negative with propionate (*r* = –0.94) and butyrate (*r* = –0.91), therefore our observations might indicate that the shift in the molar proportions of VFA induced by defaunation seems to be mainly driven by a decrease in the fiber digestion. On the basis of stoichiometry, such a shift in rumen VFA production should result in a decrease in methane production as less metabolic H_2_ will be available as a substrate for methanogenesis (Demeyer et al., 1996). The effect of defaunation on methane production is however still not clear; in various reviews compiling *in vivo* and *in vitro* studies (Hegarty, 1999) or just *in vivo* (Morgavi et al., 2010) it was concluded that removal of protozoa from the rumen would result in a 13 and 10.5% decrease in methane production, which fully agree with our results based on more recent studies (–11%, *P* = 0.074). In line with these observations, in a meta-analysis containing 28 experiments and 91 treatments, it has been reported a significant linear relationship between methane emissions and protozoal concentration (*r* = 0.96) with a decrease in methane yield averaged 8.14 g/kg DMI per each log unit reduction in the protozoal concentration (Guyader et al., 2014), suggesting that protozoa played a catalytic role in rumen methanogenesis. The reasons for the lower methane emissions in defaunated animals are still controversial since the number of rumen protozoa explains only one part (approximately 47%) of the variability in methane emissions indicating that rumen methanogenesis is a complex process in which multiple microbes are involved (Morgavi et al., 2010). A number of mechanisms by which protozoa could enhance methanogenesis are possible based on their ability to produce H_2_ in their hydrogenosomes (a mitochondria-like organelle), their ability to host epi- and endo-symbiotic methanogens and to protect them from oxygen toxicity (Fenchel and Finlay, 2006). This interaction is a typical example of interspecies H_2_ transfer that favors both the methanogens and the protozoa (Ushida et al., 1997). One hypothesis is that defaunation leads to decreased methanogen numbers, which are the sole producers of methane in the rumen, but our meta-analysis reported that this decrease in methanogens levels was not significant (–3%, *P* = 0.48). An alternative hypothesis suggests that defaunation results in the elimination of protozoa-associated methanogens, which could be considered as one of the most active methanogens communities in the rumen (Finlay et al., 1994), however this later hypothesis based on the substitution of methanogen communities which differ in their methanogenic activity requires further investigation. Several authors have studied the endo-symbiotic archaeal population in protozoa (Sharp et al., 1998; Irbis and Ushida, 2004; Regensbogenova et al., 2004b; Tymensen et al., 2012a) and most of them agree that *Methanobrevibacter* sp. is the predominant genus, while the contribution of *Methanomicrobium* sp. and *Methanomassiliicoccales* to the endosymbiotic methanogen community is variable and could indicate differences among protozoal groups. The development of molecular techniques over the last decades has allowed colleagues to further explore these microbial interactions revealing that ciliate endosymbiotic methanogens differ from rumen free-living methanogens (Tokura et al., 1997; Sharp et al., 1998). In a recent publication it has also been demonstrated that holotrich protozoa have a different endosymbiotic methanogens than entodiniomorphids (Belanche et al., 2014) possibly because either holotrich protozoa have more active hydrogenosomes than entodiniomorphids (Paul et al., 1990) and/or rapid synthesis of glycogen by holotrich protozoa in the presence of excess carbohydrates generate more hydrogen (Hall, 2011; Denton et al., 2015). These differences may explain the greater impact of holotrich protozoa on rumen methanogenesis compared to entodiniomorphids (Belanche et al., 2015). This later observation is based on the successive inoculation of fauna-free sheep with various protozoal groups, thus it should be cautiously interpreted due to possible confounding effects of treatment and period.

The effect of defaunation on the rumen microbial ecosystem is not limited to only the methanogen population and it has been demonstrated that defaunated animals had significantly greater ruminal bacterial populations (+9%, *P* < 0.001) than control animals. The reason for this seems to be based on the availability of an ecological niche for the bacteria when protozoa are not present in the rumen combined with the removal of protozoal predation (Williams and Coleman, 1992). Recent studies using molecular techniques have demonstrated that defaunation also modified the structure of the bacterial community leading to a simplification in population structure and lower bacterial diversity (Belanche et al., 2012b, 2015). Our meta-analysis revealed a drop in the concentration of fibrolytic microbes such as anaerobic fungi (–92%, *P* < 0.001), *Ruminococcus albus* (–34%, *P* < 0.001) and *Ruminococcus flavefaciens* (–22%, *P* < 0.001) in the absence of protozoa. This observation suggests that fiber digestion in the rumen is a complicated task which requires the symbiotic collaboration of several fibrolytic microbes, including rumen protozoa, to carry out the initial stages of fiber colonization and digestion; therefore the absence of rumen protozoa seems to have a detrimental effect on this fibrolytic consortium and ultimately in fiber digestion. However, Hsu et al. (1991) reported an increase in ruminal fungal zoospores in defaunated animals possibly as a result of the removal of protozoal predation and competition for nutrients (protozoa vs. fungi), clearly more studies are needed to fully understand protozoa interactions with other rumen microbes under different dietary regimes.

The effect of the presence of rumen protozoa on pathogen’s survival in the rumen and pathogen shedding is another area of interest. As noted above rumen protozoa engulf and digest a wide range of bacteria (Williams and Coleman, 1992) and can reduce the shedding of potential pathogens from the animal, although the effect is highly dependent on the composition of the protozoal population present (Stanford et al., 2010). However, it has also been shown that rumen protozoa enhance the pathogenicity of certain pathogens leaving the rumen (Rasmussen et al., 2005; Carlson et al., 2007) suggesting that more work is needed in this area.

Figure 2 summarizes the effects of protozoa on animal productivity as the indirect effect of defaunation on the rumen function. Defaunation improved feed conversion rate as a result of the lower DM intake (–2%, *P* < 0.001) and greater average daily gain (+9%, *P* < 0.001). These positive effects of defaunation are particularly obvious with poor quality diets in which the average daily gain was low, possibly as a result of the lower availability of fermentable energy and rumen digestible protein for the rumen microbes (Williams and Coleman, 1992). In a previous meta-analysis the effect of defaunation appeared more pronounced when the ratio N/NDF was below 6 and the percentage of concentrate lower than 40% (Eugène et al., 2004). As defaunated animals have lower feed digestibility, absorbed energy is lower than in conventional animals. Thus better feed conversion rate after defaunation may be mainly attributed to the higher efficiency of utilization of absorbed nutrients. Indeed our meta-analysis showed that defaunation promotes a greater efficiency of energy utilization for fattening (+11%, *P* = 0.001), possibly as a result of a lower heat production (–5%, *P* = 0.010). Other hypothesis to explain the better performance of defaunated animals rely on a more efficient use of dietary protein. There is clear evidence that holotrich protozoa leave the rumen more slowly than bacteria (Abe et al., 1981). The amount and rate of protozoal flow to the lower gut is however the subject of much debate. Firkins et al. (2007), found that post-ruminal flow of protozoa is proportional to rumen protozoal biomass. Although, protozoal counts in the rumen and abomasal fluid indicated that abomasal counts were only 6–64% of rumen fluid counts (Punia et al., 1992), numbers of protozoa in free rumen fluid are unreliable indicators of protozoal biomass and outflow, because the majority of rumen protozoa (63–90%) are found either associated with feed particles or sequestered in the rumen wall (Hook et al., 2012). The use of specific markers of protozoa such as 2-aminoethylphosphonic acid has been questioned because it is present in bacteria and in feed (Ling and Buttery, 1978). Protozoal outflow measured by use of a general microbial markers (e.g., ^15^N and ^35^S and purine bases), microbial N minus specific bacterial markers (such as 2,6-diaminopimelic acid, DAPA) yielded variable estimates of protozoal outflow and sometimes even negative values due to methodological limitations for the markers used (Broderick and Merchen, 1992). Recent studies based on the use of 18S rRNA as a novel protozoal marker have reported that although protozoa can represent up to 60% of the rumen microbial biomass, they rarely exceeds 20% of the microbial protein flow into the small intestine (Sylvester et al., 2004, 2005; Yáñez-Ruiz et al., 2006; Belanche et al., 2011, 2012b). However, these new techniques are not free from their own limitations: Sylvester et al. (2005) observed that up to 48% of the protozoal N was actually N from contaminating bacteria which could led to inflate the protozoal N pool in the rumen, particularly when animals have low feed intakes and low particulate passage rate (Dijkstra, 1994). Similarly, Belanche et al. (2010b) observed a greater degradation of protozoal DNA compared to bacterial DNA during abomasal digestion which could result in an underestimation of the protozoal N outflow from the rumen. Despite these limitations, these new findings seem to support that rumen protozoal are partially sequestrated in the rumen. This rumen sequestration is however not equal for all protozoal groups; holotrich protozoa associate to the feed particles after feeding due to their strong chemotaxis toward sugars (Diaz et al., 2014), but rapidly migrate to the ventral reticulorumen to prevent being washed out of the rumen (Karnati et al., 2007), on the contrary Entodiniomorphids also associate to feed due to their moderate chemotaxis toward glucose and peptides but do not show the same affinity to the rumen wall (Diaz et al., 2014) and thus flow out the rumen with the particulate phase (Hook et al., 2012).

Protozoa predate on bacteria as their main protein source (Williams and Coleman, 1992) and as a result, defaunation makes the rumen more efficient in terms of proteosynthesis increasing the duodenal flow of microbial protein (+30%, *P* < 0.001) and total non-ammonia N flow (+31%, *P* < 0.001). Defaunation also increased the efficiency of microbial protein synthesis (+27%, *P* = 0.008) as a result of both a better microbial proteosynthesis and a lower OM digestion. Protozoal generation time is far higher than that of bacteria, thus the energetic requirements for maintenance are higher when expressed as a ratio of protein leaving the rumen (Williams and Coleman, 1992). As a result, the presence of protozoa has a negative impact on the overall energetic efficiency of the rumen ecosystem. In addition, defaunation can also modify the composition of the rumen bacteria (Belanche et al., 2012b) and ultimately the amino acid profile of the duodenal protein supply promoting an increase in specific amino acids such as leucine, threonine and arginine (Ivan, 2009), but not lysine which is considered the main limiting amino acid in high producing animals (Hristov and Jouany, 2005). After a series of *in vivo* experiments in which fauna-free lambs were progressively inoculated with protozoal species (i.e., *Isotricha intestinalis*, *Dasytricha ruminantium*, *Polyplastron multivesiculatum*, *Epidinium ecaudatum*, *Eudiplodinium maggi*, and *Entodinium caudatum*) in different sequential orders (Ivan et al., 2000a,b; Ivan, 2009), Ivan concluded that holotrich protozoa engulf only a very small number of rumen bacteria and have a small effect on the duodenal NAN flow and protein metabolism. Similarly, holotrich protozoa had no significant effect on the fiber digestion. This together, with the contribution of holotrich’s to rumen methanogenesis (Belanche et al., 2015), seems to indicate that presence of holotrich protozoa in the rumen is of little value to ruminant production, unless high-carbohydrate diets are used, in which case the presence of holotrich protozoa could be beneficial as a result of their ability to engulf and accumulate starch grains and soluble carbohydrates (Williams and Coleman, 1992). This engulfment of highly fermentable carbohydrates prevents alternative bacterial fermentation that would otherwise decrease pH and increase the onset of lactic acid acidosis (Mackie et al., 1978). On the contrary cellulolytic protozoa (*Polyplastron*, *Epidinium*, and *Eudiplodinium*) could be beneficial to animals fed with fibrous diets. However, these fibrolytic protozoa and *Entodinium* spp. have a substantial potential to engulf and degrade bacteria (Belanche et al., 2012a) and might be detrimental in terms of protein utilization by the ruminant host. Therefore their presence in the rumen may not be helpful in animals fed low protein diets because their fibrolytic beneficial effects would be counterbalanced by the sensitivity of fibrolytic bacteria and anaerobic fungi to N shortage in the rumen (Belanche et al., 2012c). Thus it seems that defaunation decreased urinary N losses here (–10%, *P* < 0.001) due to a combination of lower dietary CP degradation in the rumen and lower rumen bacterial breakdown. However, the great variability observed in terms of N balance highlights the fact that defaunation may have different effects on the overall efficiency of N utilization depending on the diet consumed by the animal in particular the level of intake and particle passage rate through the tract (Dijkstra, 1994).

The effects of defaunation on ruminal lipid metabolism are less defined. The protozoa contribute to the total microbial lipolytic activity but their role in bio-hydrogenation is less well-understood (Williams and Coleman, 1992) although they do contribute significantly to flow of unsaturated fatty acids leaving the rumen (Yáñez-Ruiz et al., 2006). Using steers fed diets with different chlorophyll levels, it was demonstrated that the high levels of polyunsaturated fatty acids in protozoal cells appears to be associates with ingestion of chloroplasts (Huws et al., 2009). This chloroplasts uptake seems to be specific of Entodiniomorphids since no engulfed chloroplasts have been found in holotrich protozoa (Huws et al., 2012). Thus protozoa appear to protect chloroplast unsaturated fatty acids from the rumen bio-hydrogenation increasing the duodenal flows of mono and polyunsaturated fatty acids. Our meta-analysis agrees with this observation, and defaunation promoted an increase of saturated fatty acids (+4%, *P* = 0.046) and a numerical decrease in mono and polyunsaturated fatty acids (–13 and –10%, respectively) which possibly did not reached significance as a result of diet-dependent effects.

The findings reported in our meta-analysis need to be carefully interpreted since most of the studies had methodological limitations due to the intrinsic difficulty of the defaunation process leading to:

(1) Confusion between the effect of defaunation and period when using the same experimental animals in time,
(2) Nearly all studies estimating protozoal pool size have failed to report duodenal flows of protozoal biomass and *vice versa*, moreover protozoal flow is often underestimated using traditional microbial markers (Broderick and Merchen, 1992),
(3) Studies assessing methane production have not accounted for other variable effects besides protozoal abundance (i.e., NDF digestibility),
(4) Animals are often fed at levels far below the production levels of intake,
(5) In some studies the adaptation period after defaunation/refaunation is too short for the microbial ecosystem to fully adapt,
(6) Elimination of rumen protozoa modifies the ecological structure in the rumen and ultimately alternative microbial groups can take over ecological niche previously filled by protozoa.

Thus, more studies are needed using state-of-the-art technologies to quantify protozoal activity (i.e., fibrolytic and proteolytic) as well as the ruminal protozoal pool size relative to protozoal N outflow for better understanding of the role of protozoa on the ruminant’s metabolism. These studies should be done at production levels of intake in order to truly assess the effect of defaunation per unit of product (milk or meat) produced.

## Manipulation of Rumen Protozoa

Current concerns regarding the role of livestock in global warning has driven researchers to search for strategies to manipulate rumen protozoa to decrease methane production. As has been previously mentioned, there is a linear relationship between protozoal concentration and methane emissions (Guyader et al., 2014) and it has been estimated that between 9 and 37% of ruminal methane production can be attributed to methanogens associated with protozoa in the rumen (Finlay et al., 1994; Newbold et al., 1995; Machmuller et al., 2003).

Most approaches to defaunation rarely result in the total removal of protozoa from the rumen with their effectiveness largely dependent on diet composition (Hegarty, 1999). Treatments normally used to partially or completely defaunate the rumen include: chemicals that are toxic to protozoa (copper sulfate, dioctyl sodium sulfosuccinate, alcohol ethoxy-late or alkanates, calcium peroxide), ionophores, lipids, and saponins (Williams and Coleman, 1992; Jouany, 1996; Hook et al., 2010).

A recent meta-analysis by Guyader et al. (2014) has shown a concomitant reduction in protozoal numbers and methane emissions in 31% of 70 studies using different methane reduction strategies. Most of the studies used lipids as a protozoal control/methane mitigation strategy. The authors also reported that the antiprotozoal effect of lipids depends on the fatty acid composition with medium chain fatty acids more effective than polyunsaturated fatty acids in controlling protozoal numbers. Supplements rich in polyunsaturated fatty acids such as linoleic acid (C18:2 from soybean and sunflower) and linolenic acid (C18:3 from linseed) have been shown to have a negative effect on methane production (4.1 and 4.8% decrease per percentage unit of added lipids, respectively, Martin et al., 2010). The antimethanogenic effect of polyunsaturated fatty acids has been related to their toxic effect on cellulolytic bacteria (Nagaraja et al., 1997) and protozoa (Doreau and Ferlay, 1995). Although it was originally suggested that the biohydrogenation of polyunsaturated fatty acids in the rumen could represent an alternative H_2_ sink to methanogenesis (Lennarz, 1966), it is now believed that the significance of biohydrogenation to the overall H_2_ sink is small (Nagaraja et al., 1997). It has been shown that medium chain fatty acids have potent antiprotozoal effect. Several studies have reported decreased ruminal methanogenesis when supplementing lauric acid (C12:0) and myristic acid (C14:0), either in pure forms or in products rich in these fatty acids (coconut oil) under *in vitro* (Dohme et al., 2001; Soliva et al., 2004) or *in vivo* conditions (Machmuller et al., 2003; Jordan et al., 2006). Martin et al. (2010) reported that medium chain fatty acids, mainly provided by coconut oil, resulted in a decrease in methane of 7.3% per percentage unit of added lipid. However, the methane suppression effect observed was not always related to a decrease in protozoa concentration (Machmuller et al., 2003) which may be due to a direct effect of medium chain fatty acids on methanogens (Dohme et al., 1999; Panyakaew et al., 2013). A recent study (Faciola and Broderick, 2014), found that both coconut oil and lauric acid reduced the number of protozoa by 40% but whereas lauric acid altered fiber digestibility coconut oil did not. However, reductions in methane production and a concomitant decrease in dry matter intake when coconut oil and lauric acid were used as defaunating agents has been reported (Hristov et al., 2013), which would potentially limit their practical on-farm use. Clearly more studies are needed to work out how to use oils and fatty acids to control protozoa in the rumen

The literature suggests that saponins mitigate methanogenesis mainly by reducing the numbers of protozoa, whilst condensed tannins act by both reducing the number of protozoa and by a direct toxic effects on methanogens, whereas essential oils act mostly by a direct toxic effect on methanogens (Cieslak et al., 2013). In agreement with this information, a meta-analysis of the effect of phytochemicals on methanogenesis (Patra, 2010) has shown that changes in protozoa numbers followed a linear relationship with changes in methane production by saponins (*r* = 0.69) and tannins (*r* = 0.55), but this relationship was weaker (*r* = 0.45) with respect to essential oils. Methane inhibition by organosulfur compounds was not associated with changes in the protozoal population (Patra, 2010) as such compounds specifically inhibit methanogenic archaea. For tannin containing plants, the antimethanogenic activity has been attributed to the group of condensed tannins. It has been suggested that tannins have a direct effect on ruminal methanogens and an indirect effect on hydrogen production due to a reduction in fiber digestion (Tavendale et al., 2005). Regarding their effect on protozoa, some studies have reported no effect whereas others have shown a reduction in protozoa numbers in the presence of tannins (reviewed by Patra and Saxena, 2009). This inconsistency is probably due to differences in the structure and dose of the condensed tannin. Saponins, shows a more consistent inhibitory effect on rumen protozoa in the available literature. Goel and Makkar (2012) have suggested that the risk of impaired rumen function and thus reduced animal productivity is greater with tannins than with saponins and when used to decrease methane production, the effective concentration range for tannins is narrower than for saponins. Saponins are glycosylated triterpenes or steroids where the saponin is the aglycone while the glycone is a carbohydrate unit consisting of a monosaccharide or smaller oligosaccharide entity. The most commonly sources of saponins used in ruminant nutrition are *Yucca schidigera*, rich in sterol saponins (10%), and *Quillaja saponaria* which contains triterpene saponins. Lately, other sources of saponins such as tea saponins have being explored (Hu et al., 2005; Guo et al., 2008). The antiprotozoal effect of saponins is related to their interaction with the sterol moiety which is present in the membrane of protozoa (Patra and Saxena, 2009). It has been suggested that saponins with the same aglycone may have a different effect on protozoa depending on the sugar composition and arrangement (Wina et al., 2006). This effect seems to be transitory due to the cleavage of the glycosidic bond by rumen microbes (Newbold et al., 1997). The suppression of rumen protozoa by saponins or saponin-containing plants has been consistently observed in *in vitro* studies (Wina et al., 2005). However, *in vivo* studies have shown that the antiprotozoal effect of saponins tends to disappears after 7–14 days of administration (Wina et al., 2005; Patra and Saxena, 2009). It has been suggested that when saponins are deglycosylated to sapogenins by rumen microbes they become inactive (Newbold et al., 1997). Thus, it can be hypothesized that the combination of saponins with glycosidase inhibitors would avoid deglycosylation, maintaining the intact saponin and, therefore, the activity in the rumen. Preliminary *in vitro* studies (Ramos-Morales et al., 2014) using 2,5-Dihydroxymethyl-3,4-dihydroxypyrrolidine (DMDP) as a glycosidase inhibitor combined with a plant extract rich in saponins have shown the potential of this approach to maintain saponin activity over time. Another approach that is being explored by our research group is the synthesis of a chemically modified saponin without the natural glycoside bonds so the enzymatic cleavage would be structurally prohibited. The effects of saponins on rumen fermentation have not been found to be consistent. These discrepancies appear to be related to the chemical structure and dosage of saponins, diet composition, as well as adaptation of the microorganisms to saponins (Wina et al., 2005; Patra and Saxena, 2009).

Australian researchers have demonstrated the potential of vaccination against methanogens as a method for mitigating methane emissions (Wright et al., 2004). Although this technology is still developing it provides many options for long term methane reduction. Similarly, protozoa, as providers of hydrogen to methanogens, or acetogens which compete for hydrogen with methanogens, could be possible vaccine targets for the reduction of methane emissions. An immunological approach has been explored for defaunation (Williams et al., 2008). Vaccination of sheep with entodinia or mixed protozoal antigens reduced protozoa numbers and IgG antibodies generated against rumen protozoa remained active and continued to bind target cells for up to 8 h. However, no *in vivo* effect on rumen protozoa has been observed. It has been suggested that the reasons for the lack of effect may be related to insufficient amount of specific Ig delivered in saliva, need of an adjuvant to optimize the production of salivary antibodies, target other antigens of protozoa to generate a greater immune response.

## Conclusion

Since the landmark publication of the Rumen Protozoa by Williams and Coleman (1992), there has been steady but perhaps not spectacular progress in our understanding of rumen protozoa. The advent of molecular techniques has led to a raft of publications regarding protozoal diversity in the rumen and while as discussed above the techniques used have within themselves limitations in their ability to accurately quantify individual protozoal genera they have provided new insights into the diversity of ciliate protozoa in different ruminant species, in different geographies and under different dietary situations. There has been steady progress in the area of defaunation and whilst at this stage no commercially available defaunation technique has yet been marketed, it seems likely that plant extracts can be used to control protozoa in the rumen; if not completely eliminate them. Work on the consequence of elimination of protozoa has largely focused on their role in methanogenesis, reflecting current concerns regarding the role of ruminants in greenhouse gas production. However the use of meta-analysis of existing data combined with new defaunation studies have help clarify our understanding of the role of protozoa in the rumen as illustrated in Figures 1 and 2. It is however perhaps the development of molecular techniques to clone and characterize protozoal genes, originally from single species but more recently from metagenomic and transcriptomic sources, that seems to offer the greatest, but as yet largely unfulfilled, potential to help truly elucidate the role of rumen protozoa in the rumen and in the absence of progress in developing axenic culture of rumen protozoa more effort needs to be put into characterizing rumen protozoa activity through molecular methods.

## Author Contributions

All authors have equally contributed to the preparation of this publication.

### Conflict of Interest Statement

The authors declare that the research was conducted in the absence of any commercial or financial relationships that could be construed as a potential conflict of interest.
